# Locked patellar dislocation due to osteophytes: two case reports with a focused literature review

**DOI:** 10.1097/RC9.0000000000000936

**Published:** 2026-09-11

**Authors:** Ngoc Quyen Le, Trong Xuan Cuong Tran

**Affiliations:** Orthopedics, Trauma, and Rehabilitation Department, School of Medicine, University of Medicine and Pharmacy at Ho Chi Minh City, Ho Chi Minh City, Vietnam

**Keywords:** case report, intra-articular dislocation, locked patellar dislocation, osteophyte, superior patellar dislocation

## Abstract

**Introduction:**

Locked patellar dislocation is a rare injury in which the patella becomes irreducible due to mechanical obstruction. Osteophytes have been increasingly recognized as a cause of irreducibility, but the mechanisms remain incompletely characterized.

**Case presentation:**

Case 1: A 41-year-old female sustained a locked lateral, vertical, intra-articular patellar dislocation following a motorcycle collision. An inferomedial patellar osteophyte was impacted against the medial femoral condyle and entrapped within soft tissue, requiring open reduction. Case 2: A 57-year-old female presented with a superior patellar dislocation during massage. An inferior patellar osteophyte was entrapped against the femoral intercondylar notch, successfully reduced using a modified disengagement-first maneuver.

**Clinical discussion:**

Both cases demonstrate distinct osteophyte-mediated pathomechanisms of irreducibility. In both patients, imaging before definitive treatment identified the mechanical block and guided the choice between closed and open reduction. Both patients achieved excellent functional outcomes.

**Conclusion:**

Osteophytes can transform a simple patellar dislocation into an irreducible locked injury. Imaging, particularly computed tomography when indicated, can identify the mechanical block and guide safe reduction.

## Introduction

Acute patellar dislocation is a relatively common knee injury, accounting for approximately 3% of all knee injuries^[^1,2^]^. It typically occurs laterally and is readily managed with simple closed reduction. However, locked patellar dislocations are exceedingly rare^[^3–5^]^. The patella dislocates and becomes mechanically trapped – typically by rotation around its vertical or horizontal axis and/or impaction of a patellar facet or osteophyte against the lateral femoral condyle or within the trochlea^[^1,3,6,7^]^. Among these, osteophyte-related mechanical entrapment remains the least characterized, reported only in isolated cases predominantly involving superior dislocation^[^8–10^]^. Unlike the typical intra-articular vertical-rotation dislocation in young patients, in which the patella is held by a tight quadriceps mechanism and is usually reducible once the muscle is relaxed, an osteophyte introduces a rigid bony block: the prominent bone hooks against the femoral condyle or notch and, often with associated soft-tissue incarceration, converts an otherwise reducible dislocation into an irreducible, locked one. Recognizing this distinction changes management from simple closed reduction to a strategy that anticipates open or arthroscopic release.

HIGHLIGHTS

- Osteophytes can convert a simple patellar dislocation into an irreducible, locked injury.
- Case 1: an inferomedial patellar osteophyte locked a lateral, vertical, intra-articular dislocation in a 41-year-old with only mild patellofemoral osteoarthritis.
- Case 2: an inferior patellar osteophyte interlocked at the femoral intercondylar notch and was reduced by a modified disengagement-first maneuver.
- CT is not mandatory in every case, but is particularly useful when radiographs do not define the mechanical block, when an osteophyte or impaction fracture is suspected, or when closed reduction fails.
- Both patients recovered fully (Lysholm 95 and 92) with no recurrence.

We report two cases of locked patellar dislocations caused by osteophytes – one lateral vertical intra-articular dislocation and one superior dislocation – and describe a modified disengagement-first maneuver for the latter. A focused review of the relevant literature is also provided. This work has been reported in line with the SCARE 2025 criteria^[^11^]^.

## Case presentation

### Case 1

A 41-year-old female presented to the emergency department following a motorcycle collision with severe left knee pain. On physical examination, the left knee was locked in full extension. The patella was in internal vertical rotation (Fig. 1A), tender to palpation, and restricted in mobility. The knee joint showed mild effusion, and distal neurovascular examination was intact. The patient had no prior history of patellar instability or patellar dislocation.

**Figure 1.**
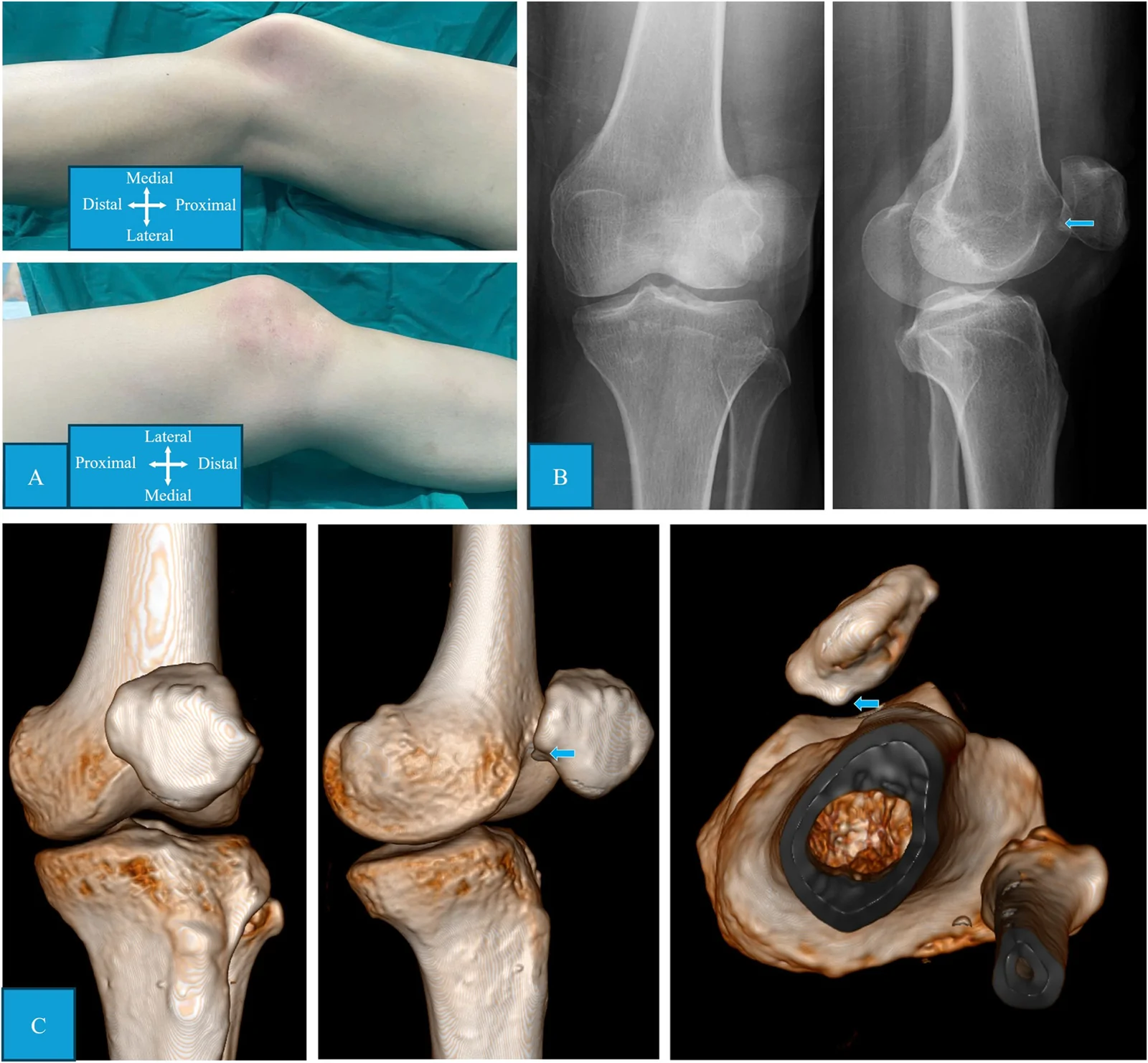
Case 1: Clinical appearance of the knee (A), radiographs (B), and 3D CT reconstructions (C) demonstrating a locked, lateral, 70° vertically rotated, intra-articular patellar dislocation. The inferomedial patellar osteophyte (blue arrow) is shown to be impacted against the medial femoral condyle, marking the point of mechanical locking.

Radiographs revealed a laterally displaced patella with vertical rotation and mild patellofemoral osteoarthritic changes, including patellar osteophytes (Fig. 1B). A skyline view could not be obtained because of severe pain. Initial attempts at closed reduction in the emergency department using an intra-articular local anesthetic were unsuccessful.

As plain radiographs did not define the cause of irreducibility, a computed tomography (CT) scan was obtained before any further intervention. CT demonstrated a locked patellar rotation of approximately 70°, with the articular surface facing laterally, and identified a definite osteophyte (4.7 × 4.3 mm) at the inferomedial pole of the patella that was impacted against the lateral edge of the medial femoral condyle. No osteochondral fractures were identified (Fig. 1C).

The patient was subsequently transferred to the operating room (8 hours after injury), where a single further gentle closed reduction attempt was performed under general anesthesia with complete muscle relaxation, with open reduction planned should it fail. This attempt was unsuccessful, and no repeated or forceful manipulation was applied. An open reduction was then performed via a midline longitudinal incision. Intra-operatively, the medial patellofemoral ligament (MPFL) was found to be completely ruptured in its mid-substance, with both the femoral and patellar insertions intact and no bony avulsion. The irreducible mechanical block was directly attributed to the inferomedial patellar osteophyte, which was impacted against the medial femoral condyle and entrapped within the adjacent soft tissue (Fig. 2A). Following careful blunt dissection to mobilize the entrapped soft tissue and disengage the osteophyte, the patella was easily reduced into the trochlear groove (Fig. 2B). The inferomedial patellar osteophyte, measuring approximately 7 × 5 mm, was not excised: it was a broad-based, rounded prominence rather than a sharp spicule; after reduction, it lay clear of the femoral trochlea and condylar articular surface throughout a full passive range of motion, with no impingement or capsular re-entrapment on repeated flexion–extension testing. Post-reduction inspection revealed no significant chondral damage, and the mid-substance MPFL rupture was repaired by direct end-to-end apposition using interrupted No. 2 non-absorbable braided sutures with the knee held at 30° of flexion. The medial retinaculum was then repaired with direct interrupted sutures along the full length of the tear, and the joint capsule was closed as a separate layer with direct suture.

**Figure 2.**
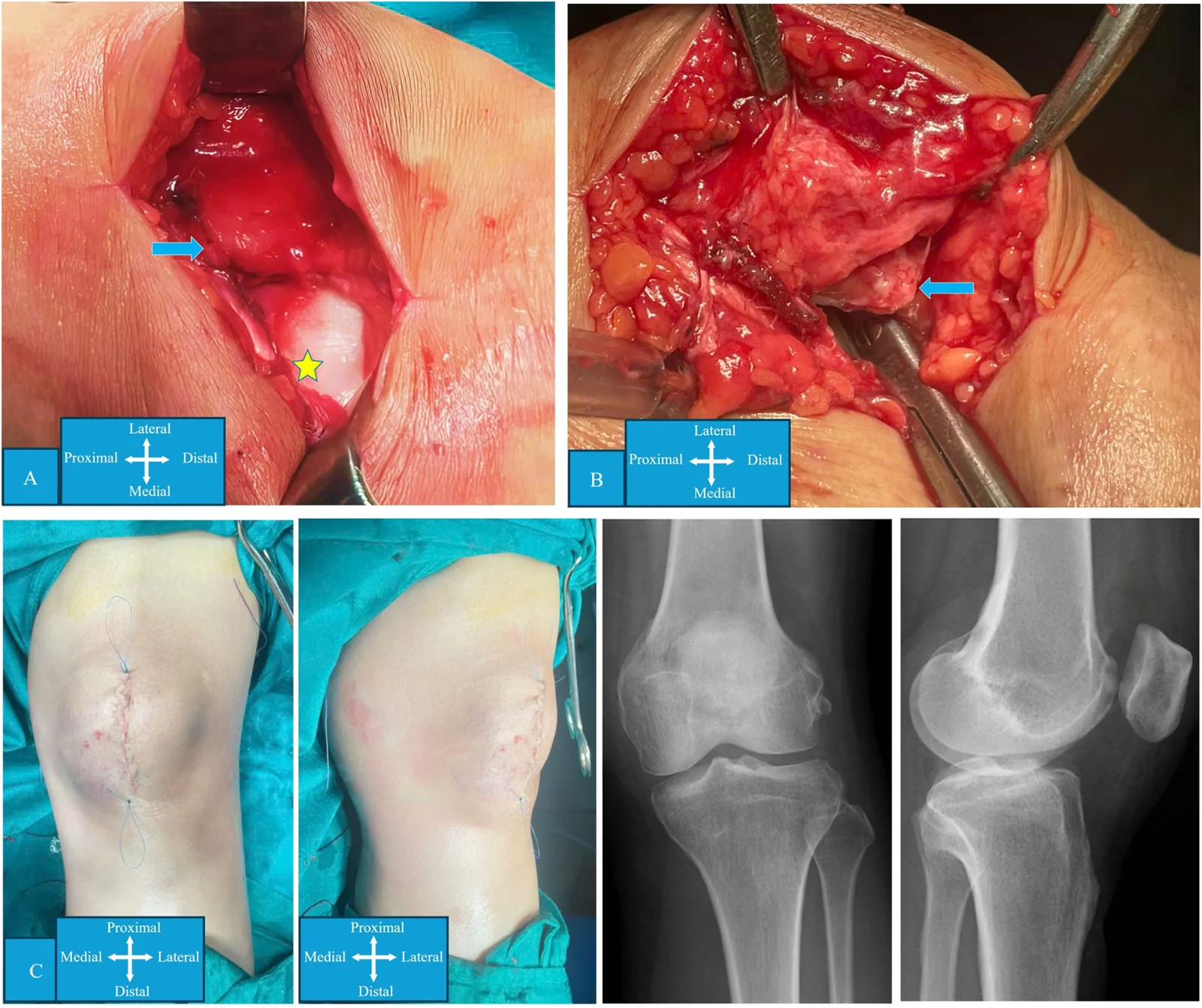
Case 1: Intra-operative photographs showing the patellar osteophyte (blue arrow) entrapped within the soft tissues and impacted against the medial femoral condyle (yellow star) – the mechanical block preventing closed reduction (A) and blunt soft-tissue release and disengagement of the osteophyte (B). Clinical appearance and postoperative radiographs of the knee showing the reduced patella with congruent patellofemoral alignment (C).

Postoperative radiographs confirmed congruent patellofemoral alignment (Fig. 2C). The patient was permitted to bear full weight with a temporary removable splint for 2 weeks and began early active range-of-motion (ROM) exercises. She was reviewed at 2, 6, and 12 weeks, then at 6 and 12 months. At the 12-month follow-up, she had a pain-free ROM (0°–130°) without extension lag. The patella tracked normally, with no clinical subluxation or redislocation. She achieved a Lysholm score of 95 and had returned fully to her pre-injury activities of daily living and work.

### Case 2

A 57-year-old female presented to the emergency department with acute right knee pain and a complete inability to flex the knee from a fully extended position. She had a history of mild bilateral degenerative joint disease (Kellgren–Lawrence grade 2) with no previous patellar instability. The injury occurred during a massage session when the practitioner applied firm, distal-to-proximal longitudinal strokes along the right pre-tibial region, resulting in sudden, severe pain and an immediate loss of motion.

Clinical evaluation revealed that the right knee was locked in full extension, with the patella superiorly displaced, which was characterized by a prominent superior pole and a visible skin dimple directly distal to the inferior pole. The patient was unable to perform a straight-leg raise, but palpation along the course of the patellar tendon demonstrated a continuous, taut tendon with no palpable gap or defect at either the inferior patellar pole or the tibial tubercle. Radiographs confirmed a superior patellar dislocation, demonstrating the inferior pole of the patella perched on the anterior aspect of the distal femur (Fig. 3A). Following an unsuccessful closed reduction in the emergency department under intra-articular anesthesia, a CT scan was performed, which revealed an inferior osteophyte (6.1 × 5.4 mm) closely abutting and incarcerated against the femoral intercondylar notch (Fig. 3B).

**Figure 3.**
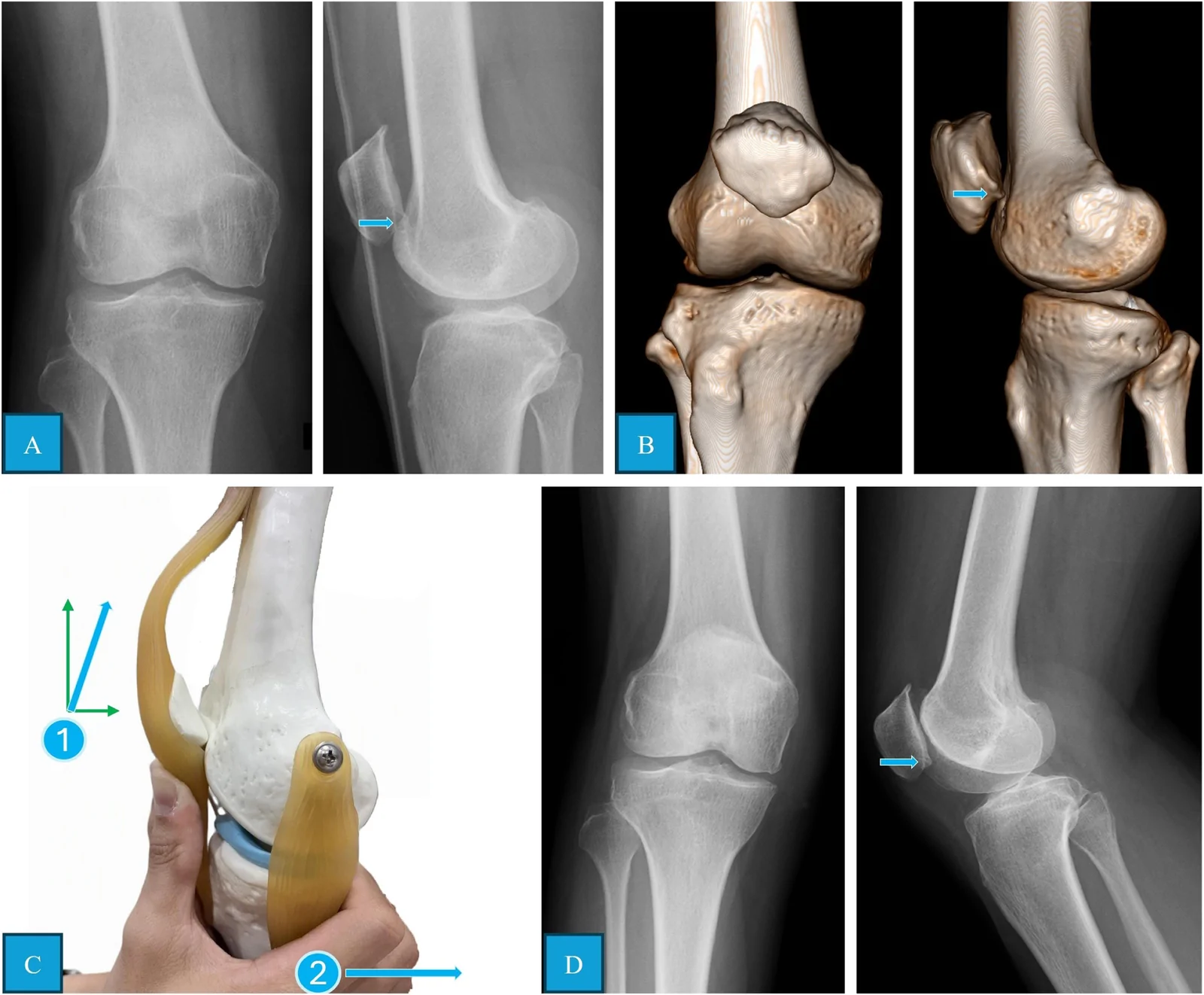
Case 2: Knee radiographs (A) and 3D CT reconstructions (B) demonstrating a locked superior patellar dislocation, with the inferior patellar osteophyte (blue arrow) locked within the femoral intercondylar notch (locking point). (C) Demonstration of the modified disengagement-first maneuver: a superiorly directed force is first applied to the inferior pole of the patella to disengage the entrapped osteophyte (step 1), followed by gentle knee flexion to relocate the patella into the trochlear groove (step 2). Postoperative radiographs (D) show the reduced patella.

The patient was taken to the operating room for management under general anesthesia (7 hours after injury). With the patient’s knee in full extension, the surgeon placed both thumbs on the inferior pole of the patella and applied gentle, controlled proximal and posterior pressure to disengage the entrapped inferior osteophyte from the intercondylar notch (step 1). Once disengagement was felt, the knee was gently flexed, allowing the patella to glide distally and relocate spontaneously into the trochlear groove (step 2) (Fig. 3C). Congruence of the patellofemoral joint was confirmed on postoperative radiographs (Fig. 3D). After reduction and recovery from anesthesia, the patient was able to perform a straight-leg raise and to achieve full active knee extension against gravity with no extensor lag, confirming an intact extensor mechanism. Postoperatively, the knee was protected in a removable knee brace, and the patient was permitted immediate full weight-bearing and discharged on the same day. Immediate active ROM exercises were begun; the brace was discontinued at 2 weeks. She was reviewed at 2 and 6 weeks, then at 3, 5, and 7 months. At the 7-month follow-up, she had a pain-free arc of motion from 0° to 135° with no extensor lag. The patella tracked normally, with no subluxation or recurrent dislocation. She achieved a Lysholm score of 92 and had resumed all normal daily activities and her occupation.

## Discussion

Locked patellar dislocation is exceedingly rare, with osteophytes increasingly recognized as a critical mechanism of irreducibility^[^3–5^]^. We present two cases of osteophyte-mediated locked patellar dislocation with distinct pathomechanisms and reduction strategies. According to the position of the patella relative to the femoral condyles^[^12^]^, dislocations are classified as intra-articular – the patella remaining anterior to the condyles but rotated about a vertical or horizontal axis (Case 1) – or extra-articular – displaced medially, laterally, inferiorly, or superiorly (Case 2).

To contextualize these cases, we searched PubMed/MEDLINE, Embase, Scopus, and Google Scholar from inception to April 2026, combining “patella” or “patellar” with “dislocation,” “locked,” “irreducible,” “intra-articular,” “superior,” and “osteophyte,” and we hand-searched the reference lists of retrieved articles. Only published case reports describing locked or irreducible patellar dislocation with sufficient clinical and imaging information to identify the mechanical locking mechanism were included. Each report was reviewed in its original publication, and duplicate reports of the same case were excluded. We identified 24 previously published case reports of locked or irreducible patellar dislocation, spanning lateral, intra-articular vertical-rotation, and superior patterns (Table 1). An osteophyte was the documented mechanical block in 8 of these cases – arising from the femoral condyle in lateral dislocations^[^3–5,13^]^ or from the inferior patellar pole in superior dislocations^[^12,14–16^]^; none described an intra-articular, vertically rotated lateral dislocation locked by a patellar osteophyte, as in our Case 1.

**Table 1:** Twenty-four previously reported cases of locked or irreducible patellar dislocation (excluding the two present cases), grouped by dislocation pattern and by the documented mechanical block.

No.	Author (year)	Age/Sex	Dislocation pattern	Mechanism	Mechanical block	Associated fracture	Reduction method	Follow-up/recurrence
**A. Lateral (extra-articular) dislocation – 15 cases**								
1	Phaltankar (2002)^[^9^]^	66/F	Lateral	Atraumatic knee torsion	Impaction fracturea	Impaction, medial patellar facet	Failed closed → arthroplasty	Immediate postoperative only; duration NR
2	Feibel (2007)^[^5^]^	66/F	Lateral	Fall on ice	Femoral osteophytea	Impaction, medial patella + lateral femoral condyle	Open (towel clip)	6 weeks; ROM 120°, no recurrence
3	Lowe (2012)^[^3^]^	50/M	Lateral with long-axis rotation	Fall walking downhill	Femoral osteophytea	Impaction, lateral femoral condyle (minor)	Open (periosteal elevator)	6 weeks; pain-free full ROM, no instability
4	Bourgeois (2018)^[^4^]^	86/F	Lateral	Fall on ice	Femoral osteophytea	Impaction, medial patellar facet	Open	1 month; patella reduced, flexion 60°
5	Kwan (2024)^[^13^]^	60/M	Lateral	Crush between vehicles	Femoral osteophytea	Fracture of medial patellar osteophyte	Closed under sedation	No follow-up available
6	Michels (2008)^[^6^]^	16/F	Lateral with vertical-axis rotation	Atraumatic (dancing)	Rotational wedginga	None	Open	1 year; no recurrence
7	Fleming (2018)^[^7^]^	15/M	Lateral with vertical-axis rotation	Direct lateral blow (football)	Rotational wedginga	None (small non-articular fragment)	Open	4 weeks; no further fragments
8	ElMaraghy (2002)^[^17^]^	30/F	Lateral with vertical-axis rotation (~180°)	Slip on ice, hyperflexion	Rotational wedging/extensor tensionb	Osteochondral fragments, medial patella + lateral condyle	Open	10 months; no instability
9	Otani (2024)^[^18^]^	22/M	Lateral, recurrent	Fall while farming	Rotational wedginga	Avulsed fragments, medial patella	Arthroscopic	5 years; no recurrence, Lysholm-equivalent good
10	Aflatooni (2023)^[^19^]^	17/M	Lateral	Pivoting (football)	Impaction fracturea	Impaction + avulsion, medial patella	Closed under sedation	No clinic follow-up at submission
11	Delagrammaticas (2016)^[^8^]^	32/F	Lateral	Twisting on planted foot	Soft-tissue interpositiona	Osteochondral fracture, medial facet	Open, lateral approach	1 year; no pain or instability
12	Teixeira (2018)^[^2^]^	23/M	Lateral	Direct high-energy blow	Soft-tissue interpositiona	None	Arthroscopic	6 months; full return to sport
13	Grewal (2016)^[^1^]^	32/F	Lateral	Direct blow (dancing)	Soft-tissue interpositionb	Avulsion, medial patellar margin	Closed under GA	2 weeks; instability on stairs
14	Balasubramanian (2019)^[^20^]^	35/M	Lateral, recurrent	Traumatic (tug of war)	Soft-tissue interpositionb	None (small chip fragments)	Closed (towel clip) under GA	Immediate postoperative only; duration NR
15	Kumar (2024)^[^21^]^	60/F	Lateral with 50–60° external rotation	Atraumatic (knee massage)	Femoral condyle impactionb	None	Closed under sedation	Patient did not attend follow-up
**B. Vertical-axis rotational dislocation (intra-articular/intercondylar) – 4 cases**								
16	Gidden (1995)^[^22^]^	15/M	Intra-articular, vertical-axis rotation	Motorcycle collision	Patella embedded in lateral condylea	Salter–Harris III, lateral femoral condyle	Open	6 months; ROM 20–100°
17	Singletary (1999)^[^23^]^	28/M	Intercondylar, vertical-axis rotation	Direct lateral blow (football)	Rotational wedginga	None	Closed under sedation	NR (referred for orthopaedic follow-up)
18	Wajid (2006)^[^24^]^	13/M	Intra-articular, vertical-axis rotation	Roller-coaster collision	Rotational wedginga	None (ipsilateral femoral shaft fracture)	Closed percutaneous (pointed reduction clamp)	~8 weeks; ROM 0–120°
19	Ahmad Khan (2016)^[^25^]^	26/M	Intercondylar, vertical-axis rotation	Missing a step	Rotational wedging/quadriceps tensiona	None	Closed under spinal anesthesia	1 year; no recurrence, normal tracking
**C. Superior dislocation – 5 cases**								
20	Siddiqui (2011)^[^14^]^	69/M	Superior	Atraumatic, while standing	Interlocking patellar osteophytea	None	Closed under sedation	NR
21	van Egmond (2017)^[^12^]^	59/F	Superior, recurrent	Unloaded hyperextension in bed	Interlocking patellar osteophytea	None	Closed, then arthroscopic osteophyte excision	12 months after arthroscopy; no recurrence
22	Dubey (2019)^[^15^]^	54/M	Superior	Direct blow on bed edge	Interlocking patellar osteophytea	None	Closed under IM analgesia	3 weeks; stable, pain-free ROM
23	Sunil (2021)^[^16^]^	58/F	Superior	Direct hit against door edge	Interlocking patellar osteophytea	None	Closed under intra-articular local anesthesia	6 months; no recurrence
24	Read (2024)^[^10^]^	65/F	Superior	Direct blow against metal beam	Not documented	None	No reduction attempted; spontaneous reduction^c^	6 weeks; no pain or instability

^a^ Block confirmed intra-operatively, arthroscopically, or on cross-sectional imaging.

^b^ Block inferred from plain radiographs, clinical findings, or the operating surgeon’s opinion, without cross-sectional or direct visual confirmation.

^c^ Reduced spontaneously without any reduction manoeuvre.

F, female; GA, general anesthesia; IM, intramuscular; M, male; NR, not reported; ROM, range of motion.

Locked lateral patellar dislocation, first described by Cooper in 1844, most often involves the medial patellar border hooked over the lateral femoral condyle (extra-articular)^[^1,3,6–8^]^. Intra-articular vertical-axis rotation has otherwise been reported only in younger patients after acute trauma^[^22,24,25^]^, with the patella wedging in the intercondylar groove from quadriceps tension rather than by a bony block^[^25^]^. Our case differs fundamentally: the inferomedial patellar osteophyte was impacted against the lateral edge of the medial femoral condyle and incarcerated by soft tissue; we emphasize this locking mechanism and its management implications rather than the primacy of the report. Notably, at 41 years of age and with mild patellofemoral degeneration, our patient was younger than the typical patient with osteophyte-mediated locked cases (usually >50 years)^[^3,5,9^]^, suggesting that even mild osteophyte formation, combined with sufficient rotational trauma, can produce irreducible intra-articular incarceration.

Management has evolved from open reduction^[^3,6^]^ toward closed reduction under conscious sedation or spinal anesthesia^[^13,19–21,23,25^]^ and, more recently, arthroscopic reduction^[^2,18^]^; in advanced tricompartmental osteoarthritis, Phaltankar and Bridle treated a locked lateral dislocation with an impaction fracture by total knee replacement^[^9^]^. In our case, one additional gentle closed reduction attempt under complete muscle relaxation, performed to exclude persistent quadriceps tension, also failed. These findings suggest that, once imaging demonstrates an osteophyte-related mechanical block, prompt open reduction should be considered, consistent with previous recommendations^[^5^]^. Our case required open reduction to free the entrapped patellar osteophyte and repair the MPFL, medial retinaculum, and joint capsule.

Locked superior patellar dislocation is even rarer. Interlocking osteophytes between the inferior patellar pole and the femoral condyle have been identified as the primary cause of locking^[^14–16^]^. An important diagnostic consideration is differentiating superior patellar dislocation from patellar tendon rupture, as both present with a high-riding patella and an inability to perform a straight leg raise. Crucially, the history distinguishes them before imaging – tendon rupture from a sudden quadriceps contraction (palpable gap, mobile patella) versus superior dislocation from trauma or hyperextension in a degenerative knee (locked patella, skin dimple) – with imaging then confirming an intact extensor mechanism and an anterior tilt of the superior pole^[^10,15,16^]^. Most reported cases have been managed successfully with closed reduction. Various closed reduction techniques have been described, including pushing the inferior pole of the patella posteriorly while pushing the superior pole distally^[^15^]^, and gentle sideways mobilization of the inferior patellar pole^[^16^]^. In our case, we hypothesized that the osteophyte became entrapped during massage as the inferior pole of the patella was pushed posteriorly and proximally, in combination with knee hyperextension. Based on this hypothesis, we performed a modified disengagement-first maneuver, deliberately exaggerating the presumed entrapment mechanism by applying pressure directed posteriorly and proximally to the inferior pole while extending the knee to disengage the osteophyte first. The knee was then flexed, allowing the patella to spontaneously relocate into the trochlear groove. Although this proposed mechanism remains speculative, the maneuver successfully achieved reduction.

Osteophytes are the key mechanical factor transforming a simple patellar dislocation into an irreducible, locked injury. CT is particularly useful in delineating the precise mechanism of locking and in guiding treatment^[^2,3,5,7^]^. Repeated, forceful closed-reduction attempts should be avoided to prevent worsening impaction fractures or chondral damage^[^1,4,5,25^]^. When closed reduction fails, open or arthroscopic intervention should be pursued promptly.

This report has several limitations. It describes only two patients and cannot establish incidence or generalizable recommendations. Follow-up was relatively short, particularly for Case 2 (7 months). Case 2 was managed by closed reduction without arthroscopic confirmation, so the osteophyte–soft-tissue mechanism is inferred from imaging rather than directly visualized. Finally, although imaging and intra-operative findings strongly implicate the osteophyte as the mechanical block, we cannot prove that the osteophyte alone, independent of associated soft-tissue factors, accounted for every step of the locking mechanism.

## Conclusion

Osteophytes can transform a simple patellar dislocation into an irreducible, locked injury through various pathomechanisms, including bony impaction, capsular tissue entrapment, and soft-tissue incarceration. Clinicians should be aware of this rare condition and, after a thorough history and clinical examination, carefully evaluate imaging findings, particularly CT, when indicated, to identify the specific mechanism of locking and guide appropriate and timely management.

## Ethical approval

Not required; written informed consent was obtained from both patients.

## Consent

Written informed consent was obtained from both patients for publication of this report and the accompanying images.

## Sources of funding

None.

## Author contributions

N.Q.L.: conceptualization, investigation, data curation, writing – review and editing. T.X.C.T.: writing – review and editing, visualization.

## Data Availability

All data are included in this article; further enquiries may be directed to the corresponding author.
